# *Capparis spinosa* improves the high fat diet-induced non-alcoholic steatohepatitis in rats: the possible role of FGF21

**DOI:** 10.1186/s13104-020-05200-4

**Published:** 2020-07-28

**Authors:** Rasoul Akbari, Hamid Yaghooti, Mohammad Taha Jalali, Laya Sadat Khorsandi, Narges Mohammadtaghvaei

**Affiliations:** 1 Hyperlipidemia Research Center, Ahvaz Jundishapur University of Medical Sciences, Ahvaz, Iran; 2 Department of Laboratory Sciences, School of Allied Medical Sciences, Ahvaz Jundishapur University of Medical Sciences, Ahvaz, Iran; 3 Cellular and Molecular Research Center, Ahvaz Jundishapur University of Medical Sciences, Ahvaz, Iran

**Keywords:** *Capparis spinosa*, Liver steatosis, Fibroblast growth factor 21

## Abstract

**Objectives:**

This study focused on the beneficial effects of *Capparis spinosa* (CS) treatment on the steatohepatitis induced by the administration of a high-fat emulsion in rats. Changes of hepatic expression and secretion of fibroblast growth factor 21 (FGF21) were also evaluated as a probable mechanism of the CS effects on fatty liver. Male Wistar rats were allocated in different groups to receive a normal diet (NC group), a high-fat diet (HF group), or the high-fat emulsion plus CS extract at a dose of 20 mg/kg (HF+CS group). Body and liver weight, liver index, serum biochemical factors, histopathological examination, and serum level and hepatic gene expression of FGF21 were determined.

**Results:**

CS administration markedly reduced liver weight and index, serum levels of glucose, lipids, alanine aminotransferase (ALT), and aspartate aminotransferase (AST) and improved histological features of nonalcoholic steatohepatitis (NASH) which were induced by HF feeding in this model. CS supplementation also restored the decreased hepatic and serum FGF21 levels in the fatty liver rats. We propose that the FGF21 up-regulation may partly account for the favorable effects of CS in this steatohepatitis model.

## Introduction

Recent studies have revealed the important role of fibroblast growth factor 21(FGF21) in the pathogenesis of non-alcoholic fatty liver disease (NAFLD) [1]. FGF21 a key metabolic regulator is predominantly produced by hepatocytes and directly modulates lipid metabolism and accumulation in the liver [2]. FGF21 reduces endogenous production of glucose, fat, and low-density lipoprotein (LDL) while promoting insulin sensitivity and utilization of fatty acids as a fuel source [3]. It has been shown that FGF21 has favorable effects in the pathogenesis of NAFLD through improving insulin resistance and alleviating hepatic steatosis, and inflammation [4]. Hence, the beneficial effects of FGF21 on insulin sensitivity and NAFLD development make FGF21 an attractive therapeutic target for the treatment of NAFLD.

Available medications for NAFLD treatment have not provided satisfactory results so far [5–10]. Therefore, it is necessary to look for novel pharmacological and/or complementary nutraceuticals for the management of the disease. Plant substances have drawn significant attention in the treatment of diseases due to large traditional beliefs, availability, and fewer side effects generally [11]. Currently, several medicinal plants and bioactive natural compounds are used to treat NAFLD in different areas of the world [12]. *Capparis spinosa* (CS) has attractive beneficial effects on diabetes and dyslipidemia [13, 14]. CS belongs to the Capparidaceae family and has widespread growth in the world as well as the southern regions of Iran [15, 16]. The favorable medical effects of CS are attributed to diverse bioactive compounds such as alkaloids and polyphenols that are present in different parts of this plant [15]. It is believed that consuming CS fruit has beneficial effects on diabetes, obesity, and cholesterol level [17–19]. It has been shown that the fruit extract of CS improved fasting blood glucose and dyslipidemia in streptozotocin-induced diabetic rats [19].

Despite these studies showing the great potentials of CS consumption on dyslipidemia, obesity, and diabetes improvement, its beneficial effects on NAFLD and their possible underlying mechanisms have not been elucidated.

The present study focused on the potential effects of CS on improving hepatic steatosis as well as glucose and lipid homeostasis. Also, the effects of CS on the hepatic expression and secretion of FGF21 were analyzed to determine a probable mechanism of action of this plant.

## Main text

### Methods

#### Preparation of the CS fruits extract

CS fruit extract was prepared and used at a dose of 20 mg/kg body weight. Fruits were deposited at the Medicinal Plant Research Center, Ahvaz Jundishapur University of Medical Sciences (Ahvaz, Iran) with the voucher number A1805812FP. Complete details of the extraction methods used were described in our previous related paper [19].

A high-fat emulsion (HF) which was consisted of corn oil 400 g, saccharose 150 g, milk powder 80 g, cholesterol 100 g, sodium deoxycholate 10 g, Tween-80 36.4 g, propylene glycol 31.1 g, vitamin mixture 2.5 g, cooking salt 10 g, mineral mixture 1.5 g, and distilled water 300 ml was prepared as described by Zou et al. [20]. Additionally, the emulsion was supplemented with vitamin and mineral mixture. The final emulsion was kept at 4 °C and before use, was heated in a 40 °C water bath and mixed completely.

#### Animal model and experimental protocol

Male Wistar rats (170 ± 20 g) were purchased from the Experimental Animal Center, Ahvaz Jundishapur University of Medical Sciences and housed under the controlled conditions of temperature (23 ± 1 °C) and lightening (12 h light/dark cycles). The rats were adapted to laboratory conditions for 7 days before the creation of the HF model. Thereafter, the rats were randomized into the following two groups: normal control group (NC group, n = 9) and high-fat model group (HF group, n = 18). The HF group was given high-fat emulsion (10 ml/kg) via gavage daily. During the study, all rats had free access to drinking water and regular chow. After 6 weeks, the rats in the HF group were allocated into the two following groups: the HF group that kept receiving high-fat emulsion and the HF+CS group that received high-fat emulsion plus CS extract at the dose of 20 mg/kg via gavage daily. At the end of the procedure at week 12, after overnight fasting, all the animals were sacrificed under anesthesia with ketamine-xylazine. Plasma samples were collected and liver samples were harvested for further assays. This study was approved by the Ethics Committee of Research Center & Experimental Animal House, Ahvaz Jundishapur University of Medical Sciences.

#### Biochemical measurements

Triglyceride (TG), total cholesterol (TC), and glucose in serum as well as plasma alanine aminotransferase (ALT) and aspartate aminotransferase (AST) activities as liver injury markers, were measured using Cobas 6000 Analyzer Series Roche (Diagnostics, Risch-Rotkreuz, Switzerland).

#### Histopathology of liver

For histopathological evaluation of hepatic steatosis, inflammation, necrosis, and fibrosis, fresh liver tissues were fixed in neutral buffered formalin and were embedded in paraffin. Then, sections of 5–7 μm thickness were cut and processed routinely for hematoxylin–eosin (H&E) and Masson trichrome staining and scoring according to the method described by Kleiner et al. [21] and Brunt et al. [22]. Briefly, hepatocellular steatosis was graded according to the percentage of hepatocytes containing macrovesicular fat (grade 0: no fat; grade 1: steatosis occupying < 33% of the hepatic parenchyma; grade 2: 34%–66%; grade 3: more than 66%). The inflammation and necrosis were graded as follows: normal—0, mild—1, moderate—2, severe—3. Hepatic fibrosis staging was determined according to the severity of injury (stage 0: none; stage 1: mild perisinusoidal or periportal; stage 2: moderate perisinusoidal or periportal; stage 3: bridging fibrosis; stage 4: cirrhosis).

#### Gene expression analysis

Total RNA was extracted from frozen liver tissue with the FastPure™ RNA Kit from Takara Bio (Otsu, Japan). RNA from each sample (5 μg) was reverse-transcribed into cDNA, using a PrimeScript RT reagent Kit (Takara Bio, Otsu, Japan). The following primers were used to determine rat FGF21 in liver tissue: forward 5′-ACCGCAGTCCAGAAAGTCTC-3′ and reverse 5′-TGCAGGCCTCAGGATCAAAG-3′. The real-time PCR was conducted using SYBR Premix Ex Taq™ II (Takara Bio, Otsu, Japan) on QuantStudio™ 3 Real-Time PCR System (ABI Applied Biosystems) following the manufacture’s instructions. The gene expression levels of FGF21 were compared with those of β-actin as a reference gene.

#### Statistical analysis

Graphpad Prism software version 8.0.2 was applied for statistical analysis. Quantitative and qualitative variables were expressed as mean ± SD or percentages, respectively. Quantitative variables were compared with one-way analysis of variance (ANOVA) and Tukey’s post hoc test. The Chi square statistic was determined for comparison of qualitative variable percentages. The significance was regarded at p ≤ 0.05.

### Results

#### Effects of CS fruit extract on changes in body weight, liver weight, and liver index

**Fig. 1:**
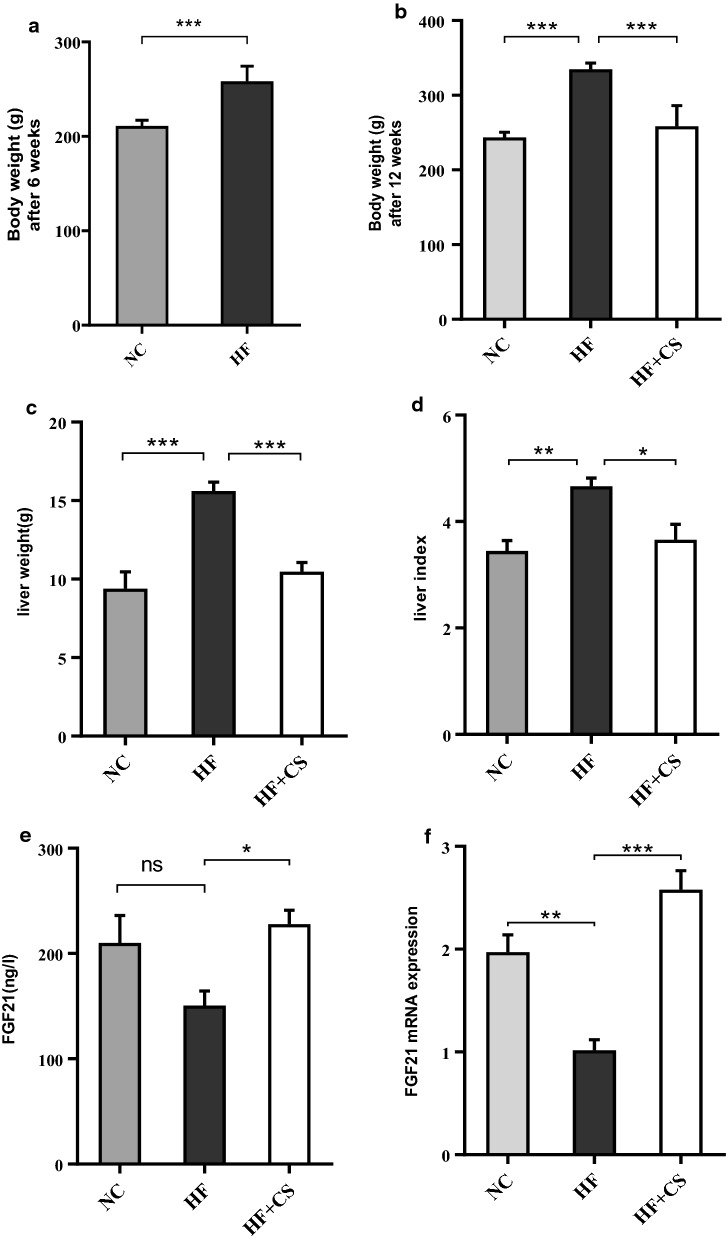
Body weight in rats of NC and HF groups after high-fat emulsion treatment for 6 weeks (**a**) effect of CS fruit extract on the body weight after 12 weeks (**b**), liver weight (**c**) and liver index (**d**) of fatty liver rats at the end of experiment. The serum concentration of FGF21 (**e**) and FGF21 mRNA expression (**f**) changes during 12 weeks of treatment. Both FGF21 concentration and its mRNA expression were significantly higher in the HF+CS group than those in the HF group. NC, normal control diet group, HF, high-fat emulsion group; HF+CS: HF + *Capparis spinosa* group. Values are given as mean ± S.D. of 7 rats at *p < 0.05; **p < 0.01; and ***p < 0.001

Changes in body weight of rats in each group were measured after 6 weeks of intervention and at the end of the experiment after 12 weeks (Fig. 1a, b). High-fat emulsion diet increased body weight in the HF group compared to the NC group after 6 weeks (p < 0.001). Treatment with CS extract significantly attenuated the body weight increase as compared to the HF group after 12 weeks (p < 0.001). As shown in Fig. 1c, d, liver weight and index of the rats were significantly reduced in the HF+CS group in response to 6 weeks CS extract treatment compared to the HF group (p < 0.001).

#### Effects of CS fruit extract on biochemical parameters

Feeding rats with the high-fat emulsion induced a striking increase in fasting blood sugar (FBS) level (+57/2%) compared with the NC group (Table 1). Treatment with CS significantly (p < 0.001) restored the rise in the FBS levels. Serum levels of TC and TG in the HF group were increased significantly (p < 0.001) as compared to the NC group. Six weeks administration of CS significantly (p < 0.001) diminished triglyceride and total cholesterol levels and alleviated NAFLD-induced dyslipidemia (Table 1). Also, rats fed with the HF-emulsion for 12 weeks showed a 1.4-fold decrease in serum FGF21 levels compared to the rats in the NC group. Treatment with CS improved the attenuated FGF21 levels in the HF+CS group (Fig. 1e).

**Table 1:** Effect of daily administration of CS (20 mg/kg) on Serum biochemistry in the experimental groups

Parameter	NC	HF	HF+CS	P Value	
				HF vs.NC	HF+CS vs. HF
TG (mg/dl)	42.57 ± 9.30	110.1 ± 11.32	49.25 ± 15.10	< 0. 001	< 0. 001
TC (mg/dl)	70.57 ± 2.63	123.1 ± 7.49	81.63 ± 8.60	< 0. 001	< 0. 001
FBS (mg/dl)	107.7 ± 7.54	123.1 ± 7.49	122.1 ± 8.72	< 0. 001	< 0. 001
ALT (IU/l)	31.57 ± 3.30	70.57 ± 5.82	40.38 ± 6.43	< 0. 001	< 0. 001
AST (IU/l)	44.71 ± 3.25	106 ± 20.07	59 ± 4.27	< 0. 001	< 0. 001

Values are expressed as the mean ± SD, n = 7. HF, high-fat emulsion group; HF+CS, HF + *Capparis spinosa* group; TG, triglyceride; TC, total cholesterol; FBS, fasting blood glucose; ALT, alanine aminotransferase; AST, aspartate aminotransferase

#### CS fruit extract alleviated HF emulsion-induced fatty liver and liver injury

**Fig. 2:**
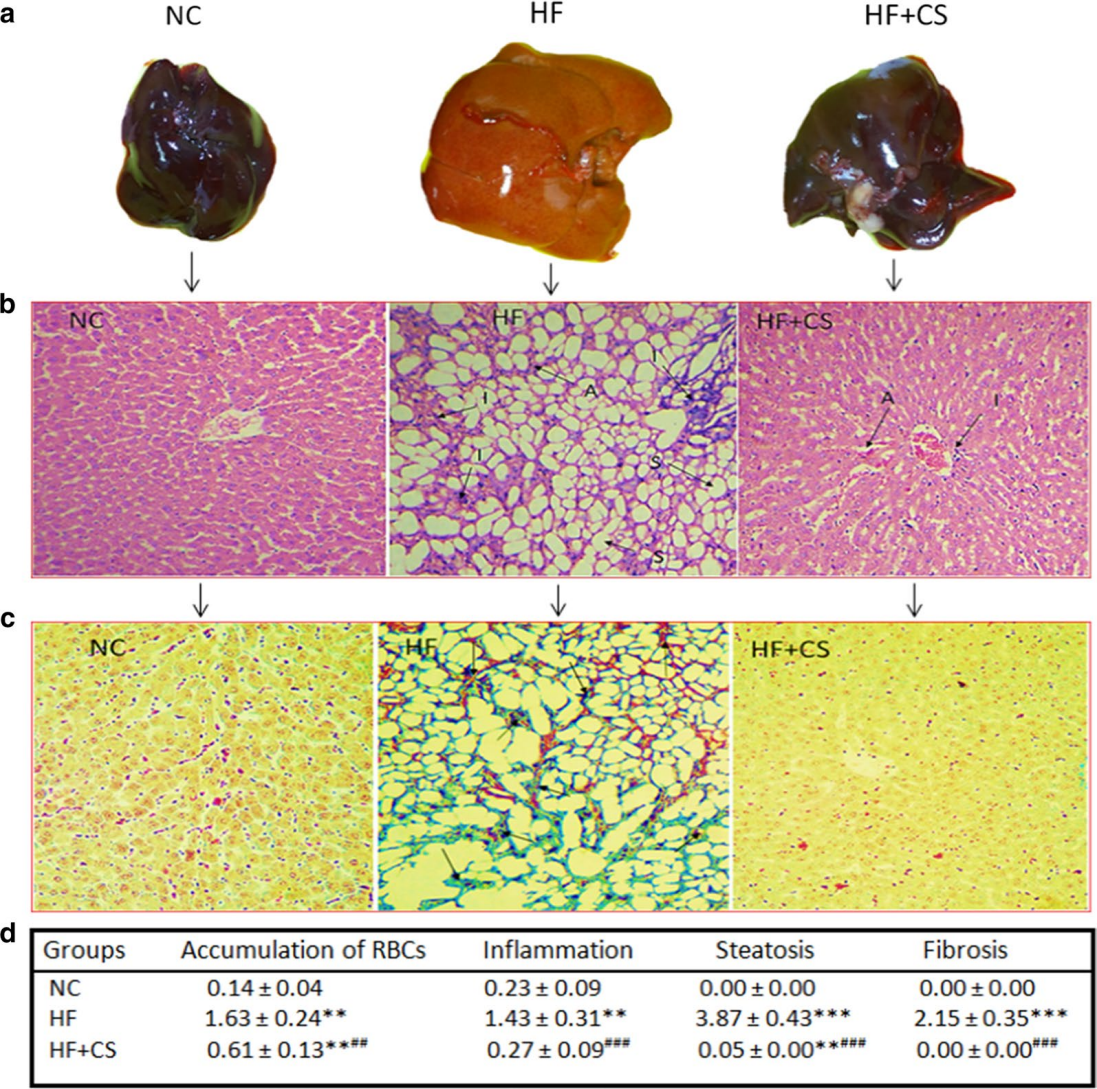
NAFLD was induced by high-fat emulsion diet. **a** Macroscopic observation of the liver of the HF+CS group showed a markedly reduced size and paler color compared with the HF group. **b** Representative images of hematoxylin–eosin and **c** Masson Trichrome stained sections of liver tissue in different groups at the end of treatments with 100X magnification. A: accumulation of RBCs; I: inflammation; S: steatosis; Arrows in c row: fibrosis. **d** Histology assessments in control and experimental groups. Values expressed as mean ± SD for 7 rats. *p < 0.05, **p < 0.01, ***p < 0.001, ^#^ p < 0.05, ^##^ p < 0.01, ^###^ p < 0.001; * and ^#^ symbols respectively indicate comparison to NC and HF groups.NC: normal control diet group; HF: high-fat emulsion group; HF+CS: HF + *Capparis spinosa* group

Serum ALT and AST levels and histopathology of the liver sections were analyzed to evaluate the effect of CS on the NASH model induced by the high-fat emulsion. Serum ALT and AST levels increased markedly in the HF group compared to the control group (Table 1, p < 0.001) which confirmed the successful establishment of the NASH model in our study. The elevated liver enzymes were significantly decreased in the HF+CS group (Table 1, p < 0.01). These findings were in parallel with histopathological alterations observed in H&E and Masson’s Trichrome staining of liver sections. H&E staining indicated that the treatment of rats with 20 mg/kg CS attenuated liver pathological injury significantly (Fig. 2) and improved liver inflammation and hepatic steatosis (Fig. 2). Masson’s Trichrome staining images for fibrosis degree are shown in Fig. 2c. No collagen deposition was found in the livers of rats on the normal diet. Livers from rats fed the HF-emulsion showed typical collagen deposition in the whole liver. The CS treatment lowered the hepatic steatosis and collagen deposition (Fig. 2d). These results indicated that CS treatment significantly improved liver injury and hepatic steatosis in the HF group rats.

#### Quantitative polymerase chain reaction (qPCR) analysis of FGF21 expression

After 12 weeks of treatment, the high fat-emulsion significantly decreased FGF 21 mRNA expression (p < 0.05) compared with the normal diet. CS extract treatment restored the hepatic FGF 21 expression (p < 0.05) compared to the HF group (Fig. 1f).

### Discussion

In the present study, we evaluated the effects of CS fruit extract on a rat model of NASH induced by a high-fat emulsion. CS administration markedly reduced serum levels of ALT and AST in this model. CS also significantly ameliorated the histological features of NASH in these rats. Our findings indicated that CS is a potentially useful herbal medicine in improving steatohepatitis induced by a high-fat diet. Additionally, given the important role of FGF21 in the regulation of metabolic pathways, we investigated the impact of CS extract on hepatic expression and serum level of FGF21. While serum and hepatic expression of FGF21 were decreased in our model, we found that CS administration up-regulated the hepatic and serum FGF21 levels.

It seems that CS fruit extract is a cocktail of effective molecules with multiple metabolic targets. Our findings suggested that the effects of CS on NAFLD improvement may be partly due to the up-regulation of FGF21 and its target metabolic pathways. The liver is the principal source of circulating FGF21 and mediates the majority of its beneficial effects on whole-body metabolism [23]. Several lines of evidence have demonstrated that transgenic overexpression or administration of FGF21 decrease hepatic and serum levels of lipids, reduce body weight, and alleviate the progression of NAFLD [1]. These are consistent with our findings as the administration of CS for 6 weeks up-regulated both hepatic and circulating FGF21 in parallel to the improvement in NASH features.

Our results indicated that CS fruit has beneficial effects on the metabolic and histologic profile of the high fat-induced NASH model in rats. Moreover, we propose that the increase in FGF21 expression and secretion in response to CS extract may partly account for these beneficial effects. These remarkable properties of CS established in the rodent model encourage future human studies to confirm the usefulness of CS in the management of fatty liver.

## Limitations

It is noteworthy that this study did not fully identify the underlying mechanisms responsible for the CS-mediated NASH amelioration, but improvements in glucose and lipid homeostasis, bodyweight reduction, and histological modification are all consistent with FGF21 as the mediator of CS actions. We also did not identify the involving signaling pathways and upstream regulators of FGF21 expression in response to CS treatment and FGF21 up-regulation.

## Data Availability

The datasets used and/or analysed during the current study are available from the corresponding author on reasonable request.

## Abbreviations

NAFLD: Non-alcoholic fatty liver disease
CS: *Capparis spinosa*
HF: High-fat
FBS: Fasting blood sugar
TG: Triglyceride
TC: Total cholesterol
ALT: Alanine aminotransferase
AST: Aspartate aminotransferase
NASH: Nonalcoholic steatohepatitis
FGF21: Fibroblast growth factor 21
